# First person – Amélie Bacle

**DOI:** 10.1242/dmm.045526

**Published:** 2020-06-17

## Abstract

First Person is a series of interviews with the first authors of a selection of papers published in Disease Models & Mechanisms, helping early-career researchers promote themselves alongside their papers. Amélie Bacle is first author on ‘A comprehensive study of phospholipid fatty acid rearrangements in metabolic syndrome: correlations with organ dysfunction’, published in DMM. Amélie conducted the research described in this article while a postdoc in Thierry Ferreira’s lab at Université de Poitiers, Poitiers, France, where she investigated lipid membranes, their composition, and the link between their properties, cellular processes and diseases, using computational techniques such as molecular modelization. She is now working in a private company developing health care applications.


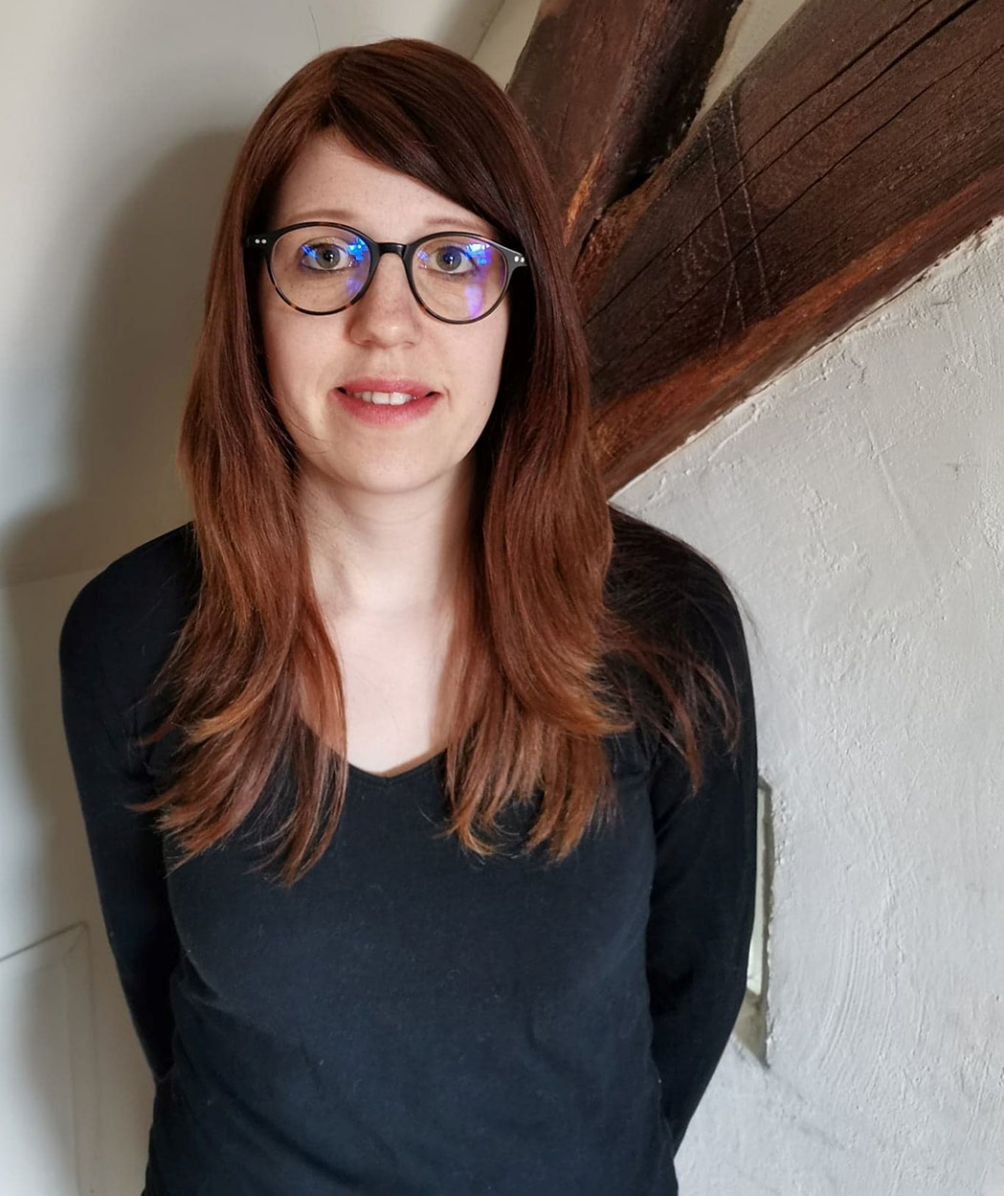


**Amélie Bacle**

**How would you explain the main findings of your paper to non-scientific family and friends?**

First, we must keep in mind that our diet constitutes an important source of fats, and more specifically of fatty acids, which are essential for the good functioning of our organs. Depending on their origin (animal or vegetal sources), these fatty acids can either be saturated or unsaturated. It has been known for more than 50 years that the Western diet, which is enriched in saturated fatty acids, is related to a cluster of metabolic diseases known as metabolic syndrome. Metabolic syndrome includes a wide range of pathologies, such as type 2 diabetes, cardiovascular disorders and hepatic steatosis.

Considering this context, we analyzed how fatty acids originating from the diet can distribute within the organs and modulate their function. With this aim, we fed some rats a normal, well-equilibrated diet, and others a diet enriched in saturated fatty acids and sugar, to mimic at best the ‘fast-food’ regimen, which is now spreading worldwide. Then, we analyzed the distribution of these fatty acids within a variety of organs (liver, brain, heart, muscles, etc). In a very surprising way, we were able to show that some organs are much more affected by food intake than others. Some organs appear to be protected from this distribution, such as the cardiovascular system, whereas others are profoundly affected, such as the liver and skeletal muscles. Accordingly, we could also show that the functions of the targeted organs are strongly impacted. Therefore, these data demonstrate that our diet can remodel some of our organs, both in terms of their biochemical composition and function.

**What are the potential implications of these results for your field of research?**

It was already known that our diet, and especially the balance between saturated and unsaturated fats, can have major impacts on our health. An important conclusion from the present study is that not all organs are equally susceptible to these consequences. This suggests that, under a chronic unbalanced diet, some protection mechanisms are at work to maintain a minimal functional state of the entire organism, by channeling the detrimental fatty acids from the diet to selective organs, and protecting others from this distribution. Of course, these results open many new perspectives in the field. For example, the implication of selective enzymes involved in fatty acid metabolism/catabolism in these protective mechanisms will have to be studied in further detail. Moreover, the molecular mechanisms accounting for the deleterious effects of saturated fatty acids in targeted organs will have to be elucidated. We were able to show that the fatty acids originating from the diet tend to distribute within specific complex lipid species, known as phospholipids. Phospholipids are the main constituents of our cellular membranes, and it is known that their fatty acid composition modulates the membrane mechanical properties. We hope that the data displayed in this publication will fuel further studies aimed at understanding to what extent such an imbalance can perturb crucial cellular properties, and if this could account, at least in part, for the etiology of the associated metabolic diseases.

**What are the main advantages and drawbacks of the model system you have used as it relates to the disease you are investigating?**

The main advantage of the model used is its reproducibility. Indeed, we could note that the lipid profile of the various organs was strongly repeatable between the two groups (i.e. rats exposed to a normal or to a high-fat, high-sugar diet). Moreover, it appears that this model recapitulates very well the situation encountered in humans. Indeed, it is well known that the liver, as the hub of fatty acid synthesis and lipid circulation through the body, is an organ for which function is altered very early in the onset of metabolic syndrome, particularly in obese individuals, who develop non-alcoholic fatty liver disease. Moreover, insulin-resistance is also an early hallmark of this syndrome, the liver and the skeletal muscle being central players in this mechanism. Finally, alterations of cardiac performance in obese individuals is attributed not only to excess body weight but also to the duration of obesity, and therefore appear in the later stages of metabolic syndrome. The model used in this study therefore recapitulates quite accurately the course of the disease in humans, with the liver and skeletal muscles being early targets and the cardiovascular system being protected, at least in the earliest stages of the disease.

**Figure:**
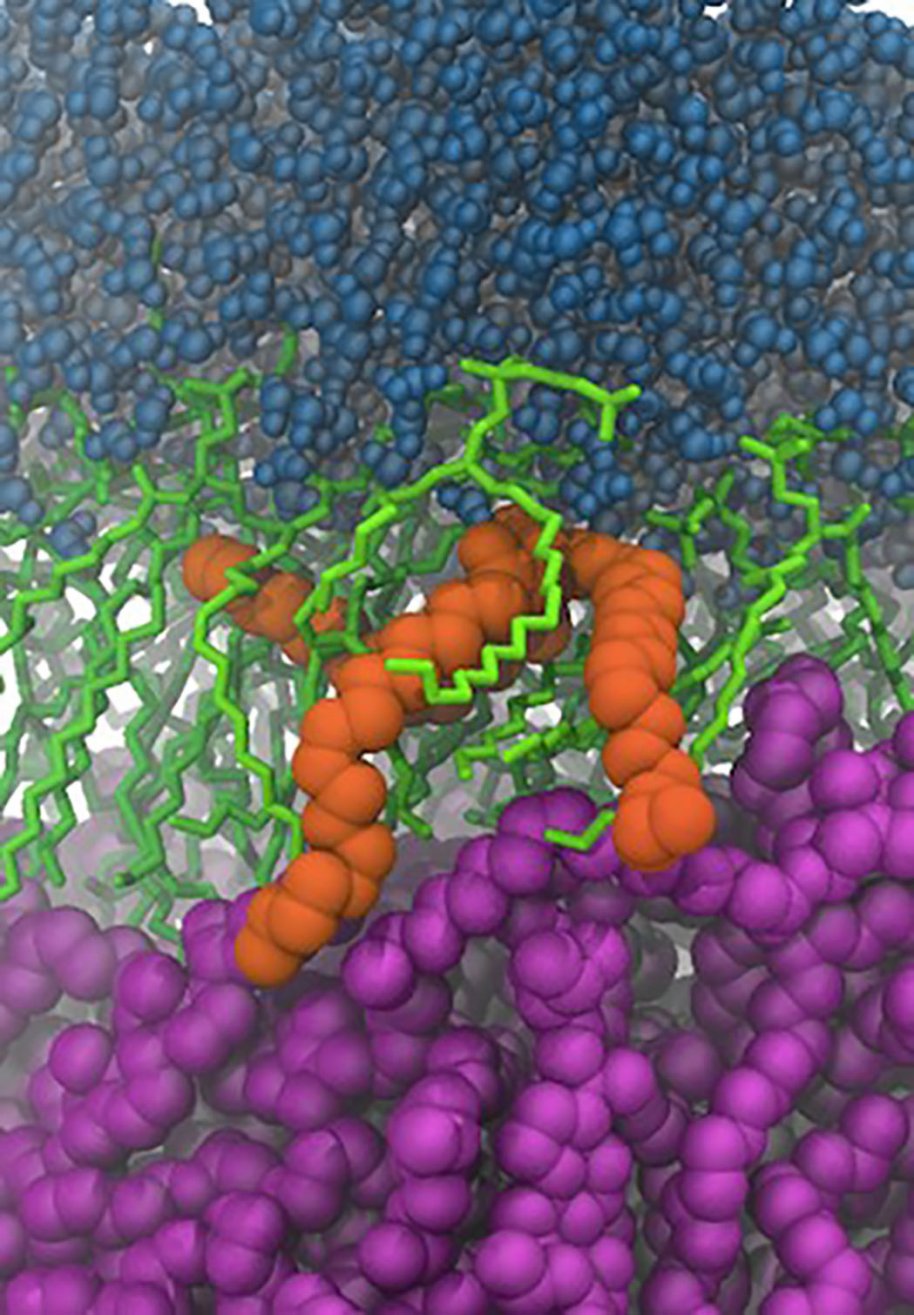
**Using *in silico* approaches, we can compute the biophysical properties of lipid membranes of various compositions, including the impacts of molecule insertions within the bilayer.**

The drawback of the model is undoubtedly its complexity, which requires experts from several different fields. However, it was a great pleasure to see how a multidisciplinary team, such as the one mobilized during the course of this study, can bring different points of views and complementary answers to the same question. This was an unprecedented and very exciting experience for us all!

“[…] it was a great pleasure to see how a multidisciplinary team, such as the one mobilized during the course of this study, can bring different points of views and complementary answers to the same question.”

**What has surprised you the most while conducting your research?**

The main surprise was to see how an imbalanced diet can profoundly affect the overall appearance and also the behavior of animals. The rats given the high-fat diet appeared to be much more aggressive than the controls. If we did not focus on these behavioral aspects in the present study, I would be very curious to know the nature of the underlying mechanisms and how they could be relevant to humans.

The second surprise came from the data concerning the cardiovascular system. Based on my reading, I was expecting the diet to have more profound effects on the heart. My thesis experience having been mainly focused on cellular and *in silico* approaches, it was very interesting to see how complex biology can be and how experimental results are difficult to anticipate when diving into the ‘*in vivo* sphere’.

**Describe what you think is the most significant challenge impacting your research at this time and how will this be addressed over the next 10 years?**

For a long time, due to the complexity of the analytical approaches required, lipids have been neglected and their role in cell function has received much less attention than other macromolecules, and particularly proteins. The emergence of mass spectrometry approaches has clearly changed the situation. The effects of lipids on membrane dynamics, and their involvement in crucial cellular processes, including vesicular budding or the folding, trafficking and function of integral proteins, are presently the subjects of intense attention. I do believe that we are at the nascent stage of this fascinating field of research. Understanding how complex lipids organize within cellular membranes and modulate their properties to match the selective processes occurring in the various compartments of the cell is very challenging. In the laboratory, we study lipids from the atomistic scale, using molecular dynamics simulations (see figure), to the cellular level. Such complementary approaches give us an integrated view of these complex processes. We also investigate the consequences of several diseases on the lipid composition of specific organs, as performed in this study, to anticipate their impacts on the biophysical properties of the targeted membranes and consequences on cell function. I sincerely believe that this strategy will lead to the identification of new candidate drugs in the future, with an innovative mode of action aiming at modulating the membrane properties in diseased cells.

**What changes do you think could improve the professional lives of early-career scientists?**

Similar to many young researchers, I am confronted by the lack of permanent positions offered in research institutions. The funding of research on the basis of ad hoc calls for projects may have positive effects in the short term, but it generates a precariousness that can push many young graduates to turn away from science. This may be very damaging in the long term.

**What's next for you?**

After a PhD and a postdoc in public labs, studying lipids using computational approaches, I'm currently working for a private company programming health care applications. I would love to go back to scientific research in a group using different approaches and techniques to study lipids and their involvement in diseases, as was the case in my previous lab. In this context, I am fully open to new opportunities.
